# Adverse Drug Reaction Study of Botulinum Toxin‐A in the Real World

**DOI:** 10.1111/jocd.70990

**Published:** 2026-06-11

**Authors:** Jiaxu Gu, Yue Sun, Kexin Chen, Jieyi Wang, Bingcheng Lu, Hongqiang Xie, Xiaoming Liu, Cong Huang, Xingling Jian, Bo Yu

**Affiliations:** ^1^ Department of Dermatology Peking University Shenzhen Hospital Shenzhen China; ^2^ Institute of Dermatology Shenzhen Peking University‐The Hong Kong University of Science and Technology Medical Center Shenzhen China; ^3^ College of Medicine Shantou University Shantou China; ^4^ Department of Dermatology Shenzhen Xinhua Hospital Shenzhen China; ^5^ Department of Intensive Care Unit Nanjing Drum Tower Hospital Clinical College of Xuzhou Medical University Nanjing China

**Keywords:** adverse event, botulinum toxin, drug reaction, FDA

## Abstract

**Background:**

Despite the increasing use of botulinum toxin type A (BoNT‐A) in aesthetic and therapeutic applications, its real‐world adverse drug reaction (ADR) profile remains incompletely characterized. Current evidence relies largely on small‐scale clinical observations rather than large, systematic analyses.

**Objective:**

To evaluate ADRs associated with BoNT‐A using pharmacovigilance data from the FDA Adverse Event Reporting System (FAERS) database (2004–2025).

**Methods:**

We analyzed 155 449 BoNT‐A‐related ADR reports from the FAERS database after deduplication and data cleaning. Disproportionality analyses were conducted using four established algorithms: reporting odds ratio (ROR), proportional reporting ratio (PRR), Bayesian confidence propagation neural network (BCPNN), and multi‐item gamma Poisson shrinker (MGPS). We evaluated time‐to‐onset and system organ class (SOC) distributions and identified high‐risk ADR signals.

**Results:**

A biphasic pattern of ADR onset was observed, with an initial peak within 30 days after injection and a second, smaller peak beyond the 360‐day mark. The most frequently reported SOCs were general disorders and administration‐site conditions (33.22%); injury, poisoning, and procedural complications (16.81%); and nervous system disorders (11.16%). Eyebrow ptosis, botulism, blepharoptosis, facial paresis, and reuse of single‐use products were identified as strong safety signals. Females accounted for 87.8% of reported cases, and the 18–64.9 years age group was the most affected.

**Conclusion:**

BoNT‐A is associated with a broad ADR profile spanning multiple organ systems, with identifiable temporal patterns. These findings highlight the importance of injection technique, dose control, and patient monitoring, particularly during both early and late post‐treatment phases. Enhanced pharmacovigilance and targeted risk management strategies are needed to improve patient safety in clinical practice.

## 1 Introduction

Botulinum toxin is a neurotoxin produced by *Clostridium botulinum* that inhibits the release of acetylcholine at neuromuscular junctions and thus causes muscle relaxation and reduces secretions from cholinergically innervated glands, particularly sweat and salivary glands [1]. Over recent years, the clinical applications of botulinum toxin have increased substantially, now encompassing aesthetic procedures such as rhytid reduction and facial contouring as well as therapeutic interventions for conditions such as blepharospasm, hemifacial spasm, cervical dystonia, and hyperhidrosis [2, 3]. This increased use, however, has been accompanied by a growing number of adverse drug reaction (ADR) reports. These adverse reactions span a broad spectrum, ranging from transient local effects, such as injection‐site pain and edema, to severe systemic events such as dyspnea and anaphylaxis, with rare fatal outcomes [4].

Monitoring ADRs is essential for medication safety [5]. Systematic analysis of pharmacovigilance data can reveal ADR patterns and also help identify reactions that pose the highest risk. Together, such insights can inform clinical decision‐making. Among botulinum toxin serotypes, type A (BoNT‐A) is the most widely used in both aesthetic and therapeutic settings and accounts for the majority of reported ADRs [6]. However, current data on ADRs resulting from botulinum toxin type A (BoNT‐A) have largely been derived from studies focusing either on small case series or on specific reaction types [7]. Large‐scale investigations that systematically analyze real‐world data, especially with regard to temporal patterns and system organ class (SOC) distributions, remain absent. Consequently, the full clinical risk profile of BoNT‐A remains poorly understood.

The US Food and Drug Administration Adverse Event Reporting System (FAERS) is a publicly accessible database of post‐marketing adverse event and medication error reports that is central to drug safety surveillance [8, 9, 10, 11, 12, 13, 14, 15]. Based on the above research, this study uses real‐world data from the FAERS to systematically evaluate the clinical safety profile of BoNT‐A. We determine time‐to‐onset, cumulative incidence at the preferred term (PT) level, and SOC distributions to define periods of elevated risk and identify organ systems requiring closer clinical monitoring and targeted attention. Our study provides clinically relevant evidence to help clinicians personalize treatment plans and optimize monitoring protocols, and to support regulatory agencies in risk assessment and mitigation.

## 2 Methods

### 2.1 Data Source

This study used adverse event (AE) reports submitted to the FAERS over 86 quarters, from the first quarter of 2004 through the second quarter of 2025. The following datasets were extracted: demographic and administrative information (DEMO), drug information (DRUG), patient outcomes (OUTC), adverse reactions (REAC), report sources (RPSR), and therapy duration (THER).

### 2.2 Data Processing

Raw data were systematically cleaned and integrated. Duplicate reports were identified and removed according to the FDA‐recommended protocol: records were sorted by PRIMARYID, CASEID, and FDA_DT (date of receipt). For records with the same CASEID, the one with the most recent FDA_DT was retained. When both CASEID and FDA_DT were identical, the record with the highest PRIMARYID was selected. To ensure all relevant cases are captured, we retrieved the standardized names for all BoNT‐A formulations (including but not limited to incobotulinumtoxinA and prabotulinumtoxinA) from the MeSH database of the National Library of Medicine. Reports identifying any BoNT‐A product as the “primary suspect” were selected for analysis. Finally, all reported PTs were standardized and mapped to their corresponding SOCs using the latest version of the Medical Dictionary for Regulatory Activities (MedDRA).

### 2.3 Signal Mining

Potential safety signals were identified using four disproportionality analysis algorithms—reporting odds ratio (ROR), proportional reporting ratio (PRR), Bayesian confidence propagation neural network (BCPNN), and multi‐item gamma Poisson shrinker (MGPS)—following previously described procedures and thresholds [16]. The higher the values obtained through these four analyses, the stronger the statistical association between BoNT‐A and an AE.

All statistical analyses were performed using the R software (version 4.3.0). A two‐tailed *p* value < 0.05 was considered statistically significant.

## 3 Results

### 3.1 Overview of the SOC Distribution of AEs

**FIGURE 1:**
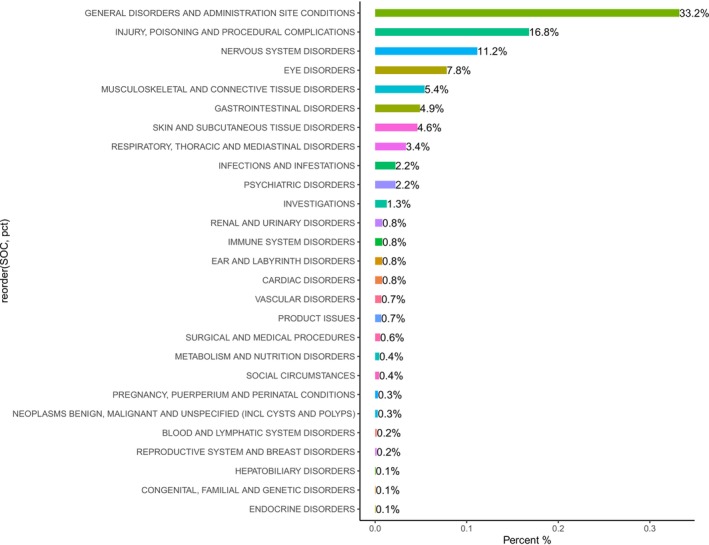
Distribution of ADRs associated with BoNT‐A by SOC.

In total, 155 449 BoNT‐A‐related AE reports, encompassing 27 distinct SOCs, were analyzed (Figure 1). The three most frequently reported SOCs were general disorders and administration‐site conditions (*n* = 51 639; 33.22%); injury, poisoning, and procedural complications (*n* = 26 128; 16.81%); and nervous system disorders (*n* = 17 342; 11.16%). Eye disorders (*n* = 12 160; 7.82%) and musculoskeletal and connective tissue disorders (*n* = 8402; 5.40%) were the other notable SOCs.

Gastrointestinal disorders (*n* = 7627; 4.91%); skin and subcutaneous tissue disorders (*n* = 7171; 4.61%); respiratory, thoracic, and mediastinal disorders (*n* = 5233; 3.37%); and psychiatric disorders (*n* = 3444; 2.22%) were other SOCs of note. Endocrine disorders and congenital, familial, and genetic disorders were rarely reported (0.06% each).

### 3.2 Clinical Baseline Features

After deduplication and data cleaning, 60 622 unique cases were retained for analysis. Baseline demographic and reporting characteristics are presented in Table 1.

**TABLE 1:** Baseline characteristics of adverse event reports associated with botulinum toxin‐A (BoNT‐A) treatments.

Category	Sub‐item	Quantity (*n*)	Proportion (%)
Sex	Female	53 208	87.8
	Male	7414	12.2
Weight	< 50 kg	932	1.5
	> 100 kg	331	0.5
	50–100 kg	4948	8.2
	Missing data	54 411	89.8
Age	< 18 years old	1244	2.1
	> 85 years old	214	0.4
	18–64.9 years old	23 001	37.9
	65–85 years old	3529	5.8
	Missing data	32 634	53.8
Occupation	Consumer	19 707	32.5
	Health professional	8401	13.9
	Other Laborer	31	0.1
	Physician	18 270	30.1
	Pharmacist	1112	1.8
	Nurse	6	0
	Other occupation	11 137	18.4
	Missing data	1958	3.2
Outcome	Congenital anomaly	8	0
	Death	1240	2
	Disability	597	1
	Hospitalization	2396	4
	Life‐threatening	285	0.5
	Recovery	39	0.1
	Other outcome	3673	6.1
	Missing data	52 384	86.4
Reporter country	United States	51 421	0.85
	Other countries	9201	0.15
Serious cases or not	Not serious	52 384	86.4
	Serious	8238	13.6
Fatal or not	Not fatal	59 382	98
	Fatal	1240	2
Reporter type	Consumer	19 707	32.5
	Health professional	8401	13.9
	Pharmacist	1112	1.8
	Physician	18 270	30.1
	Missing	13 132	21.7
Data acquisition year	2004	110	0.2
	2005	106	0.2
	2006	120	0.2
	2007	170	0.3
	2008	482	0.8
	2009	372	0.6
	2010	4476	7.4
	2011	1493	2.5
	2012	2935	4.8
	2013	2938	4.8
	2014	2889	4.8
	2015	3314	5.5
	2016	4428	7.3
	2017	4394	7.2
	2018	4839	8
	2019	5110	8.4
	2020	4156	6.9
	2021	4364	7.2
	2022	4147	6.8
	2023	5868	9.7
	2024	3911	6.5

The majority of AEs were in female patients (87.80%), a proportion roughly seven times greater than that of males (12.20%). The highest proportion of events (37.90%) was reported in the 18–64.9 years age group. Healthcare professionals submitted 45.80% of all reports, with physicians contributing 30.10%, other medical staff 13.90%, and pharmacists 1.80%. Reports submitted by patients themselves accounted for a substantial 32.50%.

Most AEs were of low severity, with 86.40% of cases classified as nonserious by the reporter and 98.00% as nonfatal. The most commonly recorded outcomes were other outcomes (6.10%), hospitalization (4.00%), and death (2.00%). Geographically, most reports (84.82%) originated from the United States of America. The annual reporting volume rose steadily over the study period until 2010, when a marked increase was observed. A modest fluctuation was observed between 2020 and 2022, followed by a new peak in 2023.

### 3.3 Timing of AEs

**FIGURE 2:**
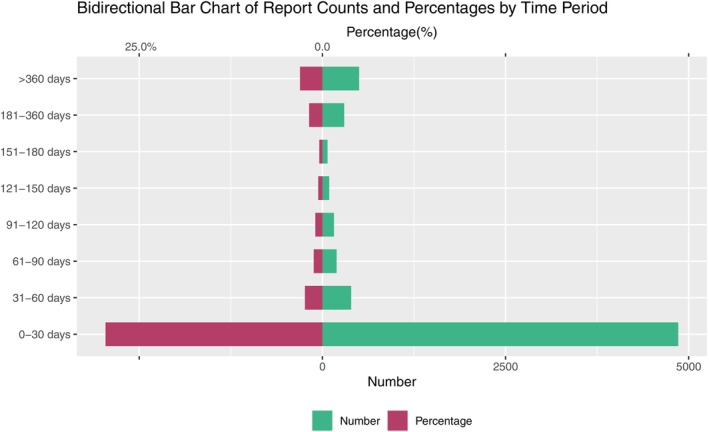
Bidirectional bar chart of BoNT‐A‐associated ADR reports by time‐to‐onset period.

Time‐to‐onset analysis revealed a biphasic distribution of event onset times (Figure 2). The first peak corresponded to AEs with onset within 30 days of injection (*n* = 4857; 29.60%). A second, smaller peak (*n* = 501; 3.0%) corresponded to AEs with onset occurring 360 days after injection. Event onset was least frequent between 151 and 180 days (*n* = 70; 0.42%).

**FIGURE 3:**
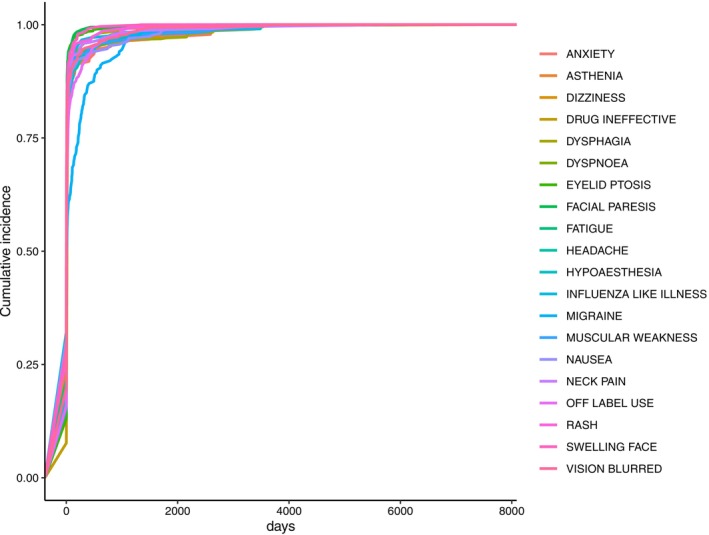
Cumulative incidence of common ADRs of BoNT‐A.

Cumulative incidence analysis identified 7347 distinct PTs (Figure 3). Dysphagia persisted for up to 7702 days, the longest documented duration. Other prolonged events included facial paralysis (6360 days), neck pain (5928 days), and drug ineffectiveness (5917 days). The majority of symptoms resolved within 4000 days.

### 3.4 AEs With Strong Disproportionality Signals

**FIGURE 4:**
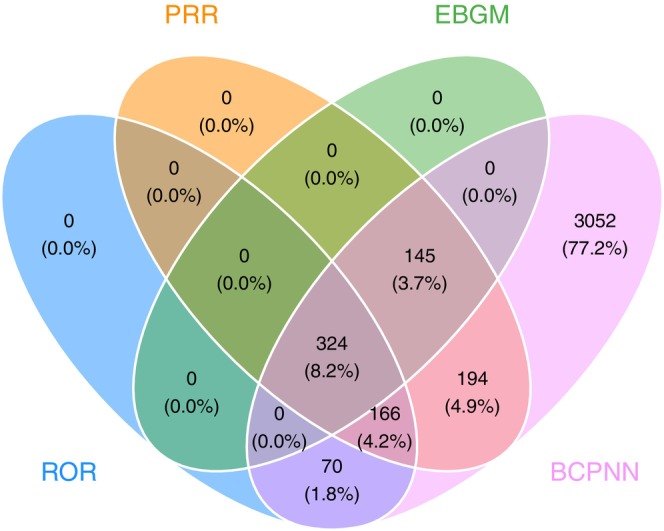
Venn diagram showing the overlap of ADR signals identified by the four pharmacovigilance algorithms.

The four disproportionality algorithms (PRR, EBGM, BCPNN, and ROR) detected 3951 safety signals at the PT level (Figure 4). Of these, 324 PTs satisfied the threshold criteria for all four algorithms, 311 met the criteria for three algorithms, and 264 for two. These findings indicate a broad range of ADRs with strong disproportionality signals.

Table 2 summarizes AEs with strong disproportionality signals across 14 SOCs and 156 corresponding PTs, identified using predefined signal detection criteria (*n* > 3, ROR > 3, and IC_0.25_ > 0). By frequency of reports, drug ineffectiveness (*n* = 22 262) and off‐label use (*n* = 11 441) were the most commonly reported PTs.

**TABLE 2:** High‐disproportionality adverse drug reactions (ADRs) associated with BoNT‐A by system organ class (SOC).

System organ class (SOC)	Preferred terms (PT)	Case reports	ROR (95% CI)	PRR (95% CI)	*χ* ^2^	IC (IC025)	EBGM (EBGM05)
General disorders and administration site conditions	Drug ineffective	22 262	8.22 (8.11–8.34)	7.19 (118 401.14)	118 401.14	2.82 (2.8)	7.05 (6.95)
Injury, poisoning and procedural complications	Off label use	11 441	6.55 (6.43–6.68)	6.14 (48 929.54)	48 929.54	2.6 (2.57)	6.05 (5.93)
General disorders and administration site conditions	Therapeutic response decreased	4340	34.1 (33.04–35.2)	33.17 (122 808.02)	122 808.02	4.91 (4.86)	30.15 (29.21)
Nervous system disorders	Headache	3501	2.2 (2.13–2.28)	2.17 (2227.19)	2227.19	1.11 (1.06)	2.17 (2.09)
Injury, poisoning and procedural complications	Product preparation error	3179	99.12 (95.23–103.17)	97.11 (231 995.87)	231 995.87	6.22 (6.13)	74.72 (71.79)
Eye disorders	Eyelid ptosis	3140	211.75 (202.39–221.54)	207.49 (391 328.68)	391 328.68	6.98 (6.86)	126.21 (120.63)
General disorders and administration site conditions	Injection site pain	3021	4.14 (3.99–4.29)	4.08 (6969.05)	6969.05	2.01 (1.96)	4.04 (3.9)
Injury, poisoning and procedural complications	Wrong technique in product usage process	2705	4.67 (4.49–4.85)	4.61 (7558.07)	7558.07	2.19 (2.13)	4.56 (4.38)
Gastrointestinal disorders	Dysphagia	1911	7.85 (7.5–8.22)	7.77 (11 013.97)	11 013.97	2.93 (2.85)	7.6 (7.27)
Musculoskeletal and connective tissue disorders	Muscular weakness	1876	6.45 (6.16–6.76)	6.39 (8376.5)	8376.5	2.65 (2.58)	6.28 (6)
Injury, poisoning and procedural complications	Multiple use of single‐use product	1450	258.89 (241.56–277.46)	256.48 (204 739.75)	204 739.75	7.16 (6.93)	142.74 (133.19)
Eye disorders	Vision blurred	1427	4.13 (3.92–4.35)	4.1 (3312.89)	3312.89	2.02 (1.94)	4.06 (3.86)
General disorders and administration site conditions	Injection site swelling	1161	6.33 (5.97–6.71)	6.29 (5073.23)	5073.23	2.63 (2.54)	6.19 (5.84)
Nervous system disorders	Migraine	1143	4.93 (4.65–5.23)	4.91 (3505.39)	3505.39	2.28 (2.19)	4.85 (4.57)
General disorders and administration site conditions	Swelling face	1121	6.78 (6.39–7.2)	6.74 (5372.7)	5372.7	2.73 (2.63)	6.62 (6.24)
Musculoskeletal and connective tissue disorders	Neck pain	1061	7.41 (6.97–7.88)	7.37 (5710.74)	5710.74	2.85 (2.75)	7.22 (6.79)
Nervous system disorders	Hypoaesthesia	966	2.44 (2.29–2.6)	2.43 (808.52)	808.52	1.27 (1.18)	2.42 (2.27)
Nervous system disorders	Facial paresis	911	133.8 (123.83–144.57)	133.02 (84 293.95)	84 293.95	6.56 (6.31)	94.22 (87.2)
General disorders and administration site conditions	Influenza like illness	850	3.85 (3.6–4.12)	3.84 (1765.29)	1765.29	1.93 (1.82)	3.8 (3.56)
General disorders and administration site conditions	Therapeutic response shortened	733	13.59 (12.62–14.63)	13.53 (8160.72)	8160.72	3.7 (3.57)	13.02 (12.09)
General disorders and administration site conditions	Drug ineffective for unapproved indication	724	5.9 (5.48–6.35)	5.87 (2877.52)	2877.52	2.53 (2.41)	5.79 (5.38)
Eye disorders	Eye swelling	713	7.67 (7.12–8.27)	7.64 (4022.33)	4022.33	2.9 (2.78)	7.49 (6.95)
Eye disorders	Diplopia	617	9.66 (8.91–10.46)	9.62 (4629.54)	4629.54	3.23 (3.09)	9.37 (8.65)
Nervous system disorders	Facial paralysis	538	15.19 (13.93–16.57)	15.14 (6786.52)	6786.52	3.86 (3.7)	14.5 (13.3)
Injury, poisoning and procedural complications	Product storage error	504	2.94 (2.69–3.21)	2.94 (637.99)	637.99	1.54 (1.41)	2.92 (2.67)
Skin and subcutaneous tissue disorders	Brow ptosis	502	10 060.91 (6115.86–16 550.72)	10 028.42 (155 469.17)	155 469.17	8.28 (7.4)	310.73 (188.89)
Respiratory, thoracic and mediastinal disorders	Dysphonia	493	3.26 (2.98–3.56)	3.25 (761.24)	761.24	1.69 (1.55)	3.23 (2.95)
Eye disorders	Dry eye	488	4.41 (4.03–4.82)	4.4 (1266.06)	1266.06	2.12 (1.98)	4.35 (3.98)
Injury, poisoning and procedural complications	Product preparation issue	487	86.99 (78.69–96.16)	86.72 (32 459.1)	32 459.1	6.1 (5.77)	68.43 (61.9)
Nervous system disorders	Burning sensation	469	2.57 (2.35–2.82)	2.57 (445.57)	445.57	1.35 (1.21)	2.55 (2.33)
Eye disorders	Eyelid oedema	466	14.95 (13.62–16.41)	14.91 (5778.25)	5778.25	3.84 (3.66)	14.29 (13.02)
Nervous system disorders	Speech disorder	460	3.29 (3–3.61)	3.29 (725.38)	725.38	1.71 (1.56)	3.26 (2.98)
Injury, poisoning and procedural complications	Poor quality product administered	439	12.3 (11.18–13.54)	12.27 (4378.16)	4378.16	3.57 (3.39)	11.86 (10.78)
General disorders and administration site conditions	Injection site rash	410	5.59 (5.07–6.16)	5.58 (1514.68)	1514.68	2.46 (2.3)	5.5 (4.99)
Eye disorders	Eye pain	375	2.84 (2.56–3.14)	2.83 (440.74)	440.74	1.49 (1.34)	2.82 (2.54)
General disorders and administration site conditions	Injection site mass	362	3.89 (3.51–4.31)	3.88 (765.78)	765.78	1.94 (1.78)	3.85 (3.47)
Injury, poisoning and procedural complications	Incorrect route of product administration	343	2.54 (2.29–2.83)	2.54 (318.01)	318.01	1.34 (1.18)	2.53 (2.27)
Renal and urinary disorders	Urinary retention	310	3.69 (3.3–4.12)	3.68 (598.84)	598.84	1.87 (1.69)	3.65 (3.26)
Skin and subcutaneous tissue disorders	Skin tightness	306	26.59 (23.66–29.88)	26.54 (6945.24)	6945.24	4.62 (4.34)	24.58 (21.88)
Skin and subcutaneous tissue disorders	Skin wrinkling	302	50.16 (44.43–56.64)	50.07 (12 556.05)	12 556.05	5.44 (5.07)	43.42 (38.46)
Infections and infestations	Botulism	296	1215.27 (946.92–1559.67)	1212.96 (74 756.23)	74 756.23	7.99 (6.87)	253.76 (197.73)
General disorders and administration site conditions	Facial pain	293	13.21 (11.75–14.84)	13.18 (3168.64)	3168.64	3.67 (3.44)	12.7 (11.3)
Nervous system disorders	Head discomfort	292	6.83 (6.08–7.67)	6.82 (1420.09)	1420.09	2.74 (2.55)	6.7 (5.96)
Musculoskeletal and connective tissue disorders	Muscle twitching	282	4.7 (4.17–5.28)	4.69 (806.91)	806.91	2.21 (2.02)	4.64 (4.12)
Ear and labyrinth disorders	Tinnitus	278	2.38 (2.11–2.68)	2.38 (220.21)	220.21	1.24 (1.06)	2.37 (2.1)
Nervous system disorders	Dysarthria	274	2.77 (2.46–3.12)	2.76 (305.87)	305.87	1.46 (1.27)	2.75 (2.44)
Musculoskeletal and connective tissue disorders	Muscle tightness	266	6.3 (5.58–7.12)	6.29 (1161.61)	1161.61	2.63 (2.42)	6.19 (5.48)
Eye disorders	Lacrimation increased	259	3.58 (3.17–4.05)	3.58 (475.81)	475.81	1.83 (1.63)	3.55 (3.14)
Pregnancy, puerperium and perinatal conditions	Pregnancy	258	4.91 (4.34–5.56)	4.91 (790.48)	790.48	2.28 (2.07)	4.85 (4.29)
Respiratory, thoracic and mediastinal disorders	Throat tightness	237	3.43 (3.02–3.9)	3.43 (403.8)	403.8	1.77 (1.56)	3.4 (2.99)
Musculoskeletal and connective tissue disorders	Facial asymmetry	235	156.72 (134.07–183.2)	156.49 (24 373.41)	24 373.41	6.72 (5.97)	105.38 (90.15)
Psychiatric disorders	Panic attack	229	2.4 (2.11–2.73)	2.4 (185.31)	185.31	1.26 (1.06)	2.39 (2.1)
Eye disorders	Photophobia	218	4.79 (4.19–5.48)	4.79 (643.66)	643.66	2.24 (2.02)	4.73 (4.14)
Eye disorders	Eye disorder	209	2.53 (2.21–2.9)	2.53 (191.53)	191.53	1.33 (1.12)	2.52 (2.2)
Gastrointestinal disorders	Lip swelling	204	2.39 (2.08–2.74)	2.38 (162.88)	162.88	1.25 (1.04)	2.37 (2.07)
Eye disorders	Periorbital swelling	201	18.64 (16.17–21.49)	18.62 (3166.54)	3166.54	4.14 (3.82)	17.65 (15.31)
Gastrointestinal disorders	Hypoaesthesia oral	197	5.06 (4.4–5.83)	5.06 (631.7)	631.7	2.32 (2.08)	5 (4.34)
General disorders and administration site conditions	Injection site urticaria	196	3.28 (2.85–3.78)	3.28 (307.27)	307.27	1.7 (1.48)	3.25 (2.83)
Injury, poisoning and procedural complications	Product administered at inappropriate site	195	3.01 (2.61–3.46)	3 (258.53)	258.53	1.58 (1.36)	2.99 (2.59)
Nervous system disorders	Paralysis	188	4.92 (4.26–5.68)	4.91 (576.97)	576.97	2.28 (2.04)	4.85 (4.2)
Eye disorders	Swelling of eyelid	172	13.69 (11.75–15.95)	13.68 (1938.05)	1938.05	3.72 (3.4)	13.16 (11.29)
Eye disorders	Asthenopia	163	12.55 (10.73–14.67)	12.53 (1664.76)	1664.76	3.6 (3.27)	12.1 (10.34)
Injury, poisoning and procedural complications	Prescribed overdose	160	3.45 (2.95–4.04)	3.45 (275.54)	275.54	1.78 (1.53)	3.42 (2.93)
Musculoskeletal and connective tissue disorders	Muscle atrophy	146	4.74 (4.02–5.58)	4.73 (423.92)	423.92	2.23 (1.95)	4.68 (3.97)
Eye disorders	Periorbital oedema	142	10.91 (9.23–12.89)	10.9 (1234.48)	1234.48	3.4 (3.06)	10.57 (8.94)
General disorders and administration site conditions	Injection site nodule	142	5.01 (4.24–5.91)	5.01 (448.4)	448.4	2.31 (2.02)	4.95 (4.19)
Nervous system disorders	Neuromuscular toxicity	140	533.2 (406.81–698.86)	532.72 (27 862.92)	27 862.92	7.65 (6.06)	200.39 (152.89)
Respiratory, thoracic and mediastinal disorders	Choking	132	2.73 (2.3–3.24)	2.73 (143.54)	143.54	1.44 (1.17)	2.72 (2.29)
Eye disorders	Blepharospasm	129	9.87 (8.28–11.76)	9.86 (996.31)	996.31	3.26 (2.91)	9.59 (8.05)
Nervous system disorders	Sensory disturbance	128	2.98 (2.5–3.55)	2.98 (166.57)	166.57	1.57 (1.29)	2.96 (2.49)
General disorders and administration site conditions	Drug effect less than expected	126	6.87 (5.76–8.2)	6.86 (618.07)	618.07	2.75 (2.43)	6.74 (5.65)
General disorders and administration site conditions	Injection site discomfort	125	5.22 (4.38–6.23)	5.22 (419.74)	419.74	2.37 (2.06)	5.15 (4.32)
Eye disorders	Ocular discomfort	122	5.18 (4.33–6.2)	5.18 (404.93)	404.93	2.35 (2.04)	5.11 (4.27)
General disorders and administration site conditions	Mass	119	3.27 (2.73–3.92)	3.27 (185.93)	185.93	1.7 (1.41)	3.25 (2.71)
Ear and labyrinth disorders	Ear discomfort	118	4.53 (3.78–5.44)	4.53 (320.34)	320.34	2.16 (1.86)	4.48 (3.74)
Musculoskeletal and connective tissue disorders	Mastication disorder	115	14.98 (12.43–18.07)	14.97 (1432.5)	1432.5	3.84 (3.41)	14.35 (11.9)
Injury, poisoning and procedural complications	Nerve injury	105	3.55 (2.93–4.3)	3.55 (190)	190	1.82 (1.5)	3.52 (2.9)
General disorders and administration site conditions	Injection site oedema	92	22.53 (18.23–27.83)	22.52 (1767.14)	1767.14	4.4 (3.81)	21.1 (17.08)
Eye disorders	Lagophthalmos	91	116.41 (91.57–147.99)	116.35 (7629.26)	7629.26	6.42 (5.14)	85.56 (67.31)
General disorders and administration site conditions	Nodule	91	2.64 (2.14–3.24)	2.63 (91.56)	91.56	1.39 (1.06)	2.62 (2.13)
Eye disorders	Eye discharge	90	3.64 (2.96–4.48)	3.64 (170.15)	170.15	1.85 (1.51)	3.61 (2.93)
Nervous system disorders	Tension headache	90	8.44 (6.84–10.4)	8.43 (574.36)	574.36	3.04 (2.63)	8.24 (6.68)
General disorders and administration site conditions	Tenderness	88	3.17 (2.57–3.91)	3.17 (129.21)	129.21	1.65 (1.31)	3.15 (2.55)
Eye disorders	Eyelid disorder	85	14.73 (11.85–18.3)	14.72 (1038.97)	1038.97	3.82 (3.3)	14.11 (11.36)
Social circumstances	Impaired driving ability	85	2.82 (2.28–3.5)	2.82 (99.25)	99.25	1.49 (1.15)	2.81 (2.27)
Nervous system disorders	Drooling	83	5.07 (4.08–6.3)	5.07 (266.65)	266.65	2.32 (1.94)	5 (4.03)
General disorders and administration site conditions	Injection site inflammation	80	6.63 (5.31–8.27)	6.62 (374.31)	374.31	2.7 (2.28)	6.51 (5.22)
Musculoskeletal and connective tissue disorders	Muscle disorder	78	3.58 (2.87–4.48)	3.58 (143.59)	143.59	1.83 (1.46)	3.55 (2.84)
Eye disorders	Ophthalmoplegia	77	15.41 (12.26–19.37)	15.4 (989.28)	989.28	3.88 (3.31)	14.74 (11.73)
Nervous system disorders	Hypotonia	76	2.93 (2.33–3.67)	2.93 (95.47)	95.47	1.54 (1.17)	2.91 (2.32)
Eye disorders	Eyelid sensory disorder	73	218.17 (162.01–293.78)	218.07 (9376.29)	9376.29	7.02 (5.17)	130.03 (96.56)
General disorders and administration site conditions	Sensation of foreign body	73	3.57 (2.83–4.5)	3.57 (133.47)	133.47	1.82 (1.44)	3.54 (2.81)
Eye disorders	Eye movement disorder	73	3.66 (2.9–4.61)	3.66 (139.36)	139.36	1.86 (1.47)	3.63 (2.88)
Respiratory, thoracic and mediastinal disorders	Aspiration	71	2.62 (2.07–3.3)	2.62 (70.28)	70.28	1.38 (1.01)	2.6 (2.06)
Eye disorders	Abnormal sensation in eye	71	6.28 (4.97–7.95)	6.28 (309.21)	309.21	2.63 (2.18)	6.18 (4.89)
Musculoskeletal and connective tissue disorders	Trismus	70	5.33 (4.21–6.75)	5.33 (242.2)	242.2	2.39 (1.97)	5.26 (4.15)
Product issues	Product distribution issue	69	5.27 (4.15–6.69)	5.27 (234.78)	234.78	2.38 (1.95)	5.2 (4.1)
General disorders and administration site conditions	Injection site hypoaesthesia	68	21.28 (16.64–27.2)	21.27 (1231.45)	1231.45	4.32 (3.61)	20 (15.65)
Social circumstances	Patient dissatisfaction with treatment	68	23.41 (18.3–29.94)	23.4 (1358.43)	1358.43	4.45 (3.71)	21.87 (17.09)
Nervous system disorders	Facial spasm	67	24.32 (18.97–31.18)	24.31 (1391.58)	1391.58	4.5 (3.74)	22.66 (17.68)
Eye disorders	Strabismus	67	9.54 (7.48–12.16)	9.53 (497.06)	497.06	3.22 (2.69)	9.29 (7.28)
Gastrointestinal disorders	Lip disorder	66	13.44 (10.51–17.2)	13.44 (729.11)	729.11	3.69 (3.1)	12.93 (10.11)
Injury, poisoning and procedural complications	Eye contusion	63	10.89 (8.47–14.01)	10.89 (547.27)	547.27	3.4 (2.83)	10.56 (8.22)
Nervous system disorders	Myasthenia gravis	61	4.25 (3.3–5.47)	4.25 (149.5)	149.5	2.07 (1.63)	4.21 (3.27)
Nervous system disorders	Muscle contractions involuntary	58	6.26 (4.82–8.11)	6.25 (251.14)	251.14	2.62 (2.12)	6.15 (4.75)
Musculoskeletal and connective tissue disorders	Posture abnormal	58	6.85 (5.28–8.89)	6.85 (283.75)	283.75	2.75 (2.24)	6.73 (5.19)
General disorders and administration site conditions	Injection site paraesthesia	55	15.81 (12.06–20.73)	15.81 (727.04)	727.04	3.92 (3.2)	15.11 (11.53)
Investigations	General physical condition abnormal	53	3.02 (2.31–3.96)	3.02 (71.06)	71.06	1.59 (1.14)	3 (2.29)
Ear and labyrinth disorders	Hyperacusis	52	5.4 (4.1–7.1)	5.4 (183.17)	183.17	2.41 (1.9)	5.32 (4.05)
Skin and subcutaneous tissue disorders	Skin mass	51	2.78 (2.11–3.66)	2.78 (57.42)	57.42	1.46 (1.01)	2.76 (2.09)
General disorders and administration site conditions	Facial discomfort	49	48.06 (35.59–64.89)	48.04 (1962.2)	1962.2	5.39 (4.09)	41.9 (31.03)
Musculoskeletal and connective tissue disorders	Torticollis	49	9.44 (7.11–12.55)	9.44 (359.18)	359.18	3.2 (2.57)	9.2 (6.92)
Nervous system disorders	Bell's palsy	45	15.5 (11.49–20.91)	15.5 (582.16)	582.16	3.89 (3.07)	14.83 (10.99)
Respiratory, thoracic and mediastinal disorders	Paranasal sinus discomfort	45	4.95 (3.69–6.64)	4.95 (139.6)	139.6	2.29 (1.74)	4.89 (3.64)
Eye disorders	Erythema of eyelid	44	3.3 (2.45–4.44)	3.3 (69.66)	69.66	1.71 (1.21)	3.27 (2.43)
General disorders and administration site conditions	Injection site hypersensitivity	44	6.82 (5.06–9.19)	6.82 (213.86)	213.86	2.74 (2.14)	6.7 (4.97)
General disorders and administration site conditions	Injection site vesicles	43	2.9 (2.14–3.91)	2.9 (52.88)	52.88	1.53 (1.03)	2.88 (2.13)
Product issues	Product temperature excursion issue	43	8.46 (6.25–11.45)	8.46 (275.49)	275.49	3.05 (2.39)	8.27 (6.11)
Skin and subcutaneous tissue disorders	Skin indentation	42	42.49 (30.8–58.63)	42.48 (1501.68)	1501.68	5.23 (3.88)	37.62 (27.26)
General disorders and administration site conditions	Injection site indentation	42	9.86 (7.25–13.4)	9.86 (324.24)	324.24	3.26 (2.55)	9.59 (7.06)
General disorders and administration site conditions	Injection site papule	39	3.24 (2.36–4.44)	3.24 (59.63)	59.63	1.68 (1.15)	3.21 (2.34)
Nervous system disorders	Guillain‐barre syndrome	39	3.39 (2.47–4.65)	3.39 (65)	65	1.75 (1.21)	3.36 (2.45)
Eye disorders	Lid sulcus deepened	39	40.22 (28.83–56.12)	40.21 (1324.59)	1324.59	5.16 (3.78)	35.83 (25.68)
Injury, poisoning and procedural complications	Occupational exposure to product	38	3.02 (2.19–4.16)	3.02 (50.89)	50.89	1.59 (1.05)	3 (2.18)
Eye disorders	Eyelid function disorder	37	18.66 (13.39–25.99)	18.65 (584.09)	584.09	4.14 (3.14)	17.68 (12.69)
Product issues	Suspected product quality issue	36	11.52 (8.26–16.07)	11.52 (333.76)	333.76	3.48 (2.65)	11.15 (8)
Nervous system disorders	Vocal cord paralysis	36	7.81 (5.61–10.87)	7.81 (208.56)	208.56	2.93 (2.22)	7.64 (5.49)
Nervous system disorders	Monoplegia	35	3.06 (2.19–4.26)	3.05 (47.92)	47.92	1.6 (1.04)	3.04 (2.18)
Infections and infestations	Injection site infection	35	4.11 (2.94–5.74)	4.11 (81.27)	81.27	2.02 (1.42)	4.07 (2.92)
Eye disorders	Eyelid pain	35	8.1 (5.79–11.32)	8.09 (212.27)	212.27	2.99 (2.24)	7.92 (5.66)
Renal and urinary disorders	Bladder pain	31	3.83 (2.69–5.46)	3.83 (64.15)	64.15	1.93 (1.29)	3.8 (2.67)
Injury, poisoning and procedural complications	Mephisto sign	31	3303.51 (1009.93–10 805.92)	3302.85 (9028.81)	9028.81	8.19 (4.14)	292.34 (89.37)
Musculoskeletal and connective tissue disorders	Temporomandibular pain and dysfunction syndrome	30	3.68 (2.57–5.27)	3.68 (57.88)	57.88	1.87 (1.23)	3.65 (2.55)
Eye disorders	Dark circles under eyes	29	7.61 (5.26–10.99)	7.6 (162.47)	162.47	2.9 (2.08)	7.45 (5.16)
Respiratory, thoracic and mediastinal disorders	Pharyngeal hypoaesthesia	28	11.87 (8.14–17.31)	11.87 (268.73)	268.73	3.52 (2.53)	11.48 (7.87)
Product issues	Suspected counterfeit product	28	3.33 (2.29–4.83)	3.33 (45.14)	45.14	1.72 (1.07)	3.3 (2.28)
Eye disorders	Eyelids pruritus	28	3.75 (2.59–5.45)	3.75 (55.92)	55.92	1.9 (1.23)	3.72 (2.56)
Eye disorders	Hypoaesthesia eye	27	19.84 (13.45–29.27)	19.84 (454.79)	454.79	4.23 (2.96)	18.74 (12.7)
Renal and urinary disorders	Bladder spasm	27	5.84 (3.99–8.55)	5.84 (106.44)	106.44	2.53 (1.75)	5.76 (3.93)
Eye disorders	Eye oedema	26	4.14 (2.81–6.1)	4.14 (61.15)	61.15	2.04 (1.32)	4.1 (2.78)
Nervous system disorders	Reduced facial expression	25	7.05 (4.75–10.48)	7.05 (127.06)	127.06	2.79 (1.92)	6.92 (4.66)
Investigations	Residual urine volume increased	23	100.72 (63.03–160.94)	100.71 (1726.5)	1726.5	6.26 (3.55)	76.82 (48.07)
Musculoskeletal and connective tissue disorders	Muscle hypertrophy	23	28.72 (18.75–44.01)	28.72 (564.57)	564.57	4.72 (3.07)	26.43 (17.25)
General disorders and administration site conditions	Therapeutic response delayed	23	3.39 (2.25–5.12)	3.39 (38.43)	38.43	1.75 (1.02)	3.37 (2.23)
Nervous system disorders	Paresis	23	3.8 (2.52–5.74)	3.8 (46.96)	46.96	1.91 (1.16)	3.77 (2.5)
Respiratory, thoracic and mediastinal disorders	Nasal oedema	23	5.34 (3.53–8.06)	5.33 (79.69)	79.69	2.4 (1.57)	5.26 (3.49)
Nervous system disorders	Tongue paralysis	22	12.1 (7.91–18.53)	12.1 (215.91)	215.91	3.55 (2.38)	11.7 (7.64)
General disorders and administration site conditions	Injection site atrophy	22	5.03 (3.3–7.67)	5.03 (70.01)	70.01	2.31 (1.48)	4.97 (3.26)
Skin and subcutaneous tissue disorders	Cutis laxa	21	28.57 (18.28–44.64)	28.56 (512.74)	512.74	4.72 (2.97)	26.3 (16.83)
Respiratory, thoracic and mediastinal disorders	Sinus pain	21	3.88 (2.53–5.97)	3.88 (44.44)	44.44	1.94 (1.15)	3.85 (2.5)
Eye disorders	Dermatochalasis	21	63.93 (40.02–102.15)	63.93 (1083.98)	1083.98	5.74 (3.31)	53.44 (33.45)
Product issues	Product reconstitution quality issue	20	11.18 (7.16–17.46)	11.18 (179.05)	179.05	3.44 (2.24)	10.83 (6.94)
Nervous system disorders	Electric shock sensation	20	4.05 (2.61–6.3)	4.05 (45.43)	45.43	2.01 (1.18)	4.02 (2.58)
Product issues	Manufacturing product shipping issue	19	15.03 (9.49–23.82)	15.03 (237.7)	237.7	3.85 (2.45)	14.4 (9.09)
Surgical and medical procedures	Face lift	19	195.93 (110.68–346.83)	195.9 (2284.24)	2284.24	6.93 (3.35)	121.84 (68.83)
General disorders and administration site conditions	Induration	19	4.06 (2.58–6.38)	4.06 (43.26)	43.26	2.01 (1.15)	4.02 (2.56)
General disorders and administration site conditions	Injection site scab	19	8 (5.08–12.62)	8 (113.56)	113.56	2.97 (1.89)	7.83 (4.97)
Gastrointestinal disorders	Tongue movement disturbance	18	8.51 (5.33–13.59)	8.51 (116.22)	116.22	3.06 (1.91)	8.32 (5.21)
General disorders and administration site conditions	Injection site anesthesia	17	19.91 (12.2–32.49)	19.9 (287.33)	287.33	4.23 (2.54)	18.8 (11.51)
General disorders and administration site conditions	Injection site granuloma	17	33.96 (20.6–56)	33.96 (491.6)	491.6	4.94 (2.82)	30.8 (18.68)
Nervous system disorders	Head titubation	17	4.78 (2.96–7.72)	4.78 (50.05)	50.05	2.24 (1.28)	4.72 (2.93)
Nervous system disorders	Occipital neuralgia	16	12.09 (7.34–19.92)	12.09 (156.83)	156.83	3.55 (2.13)	11.69 (7.09)
Nervous system disorders	Dropped head syndrome	16	18.73 (11.32–31.02)	18.73 (253.71)	253.71	4.15 (2.44)	17.75 (10.72)
Injury, poisoning and procedural complications	Iatrogenic injury	16	4.54 (2.77–7.44)	4.54 (43.57)	43.57	2.17 (1.19)	4.49 (2.74)
Eye disorders	Lacrimation decreased	15	12.33 (7.36–20.65)	12.33 (150.3)	150.3	3.57 (2.09)	11.9 (7.11)
Nervous system disorders	Facial nerve disorder	14	12.26 (7.19–20.91)	12.26 (139.43)	139.43	3.57 (2.02)	11.84 (6.94)
General disorders and administration site conditions	Injection site pallor	14	20.81 (12.12–35.74)	20.81 (247.91)	247.91	4.29 (2.36)	19.6 (11.42)
Metabolism and nutrition disorders	Marasmus	14	4.32 (2.55–7.32)	4.32 (35.24)	35.24	2.1 (1.06)	4.28 (2.52)
Nervous system disorders	Myasthenic syndrome	14	7.89 (4.64–13.41)	7.89 (82.23)	82.23	2.95 (1.66)	7.73 (4.55)
Infections and infestations	Mediastinitis	14	9.79 (5.75–16.67)	9.79 (107.23)	107.23	3.25 (1.84)	9.53 (5.6)
General disorders and administration site conditions	Therapeutic product ineffective for unapproved indication	13	13.54 (7.77–23.58)	13.53 (144.78)	144.78	3.7 (2.02)	13.03 (7.48)
Eye disorders	Periorbital pain	13	7.87 (4.54–13.64)	7.87 (76.09)	76.09	2.95 (1.6)	7.7 (4.44)
Gastrointestinal disorders	Palatal disorder	13	8.08 (4.66–14.02)	8.08 (78.71)	78.71	2.98 (1.62)	7.91 (4.56)
Investigations	Corneal reflex decreased	12	12.18 (6.84–21.67)	12.18 (118.59)	118.59	3.56 (1.87)	11.77 (6.61)
Eye disorders	Eyelid thickening	12	15.22 (8.53–27.16)	15.22 (152.19)	152.19	3.87 (2.01)	14.57 (8.17)
General disorders and administration site conditions	Injection site cyst	12	16.53 (9.25–29.54)	16.53 (166.51)	166.51	3.98 (2.06)	15.77 (8.83)
Eye disorders	Scleral hyperaemia	12	5.96 (3.36–10.54)	5.96 (48.58)	48.58	2.55 (1.28)	5.87 (3.31)
Eye disorders	Accommodation disorder	12	6.23 (3.52–11.03)	6.23 (51.64)	51.64	2.62 (1.33)	6.13 (3.46)
Skin and subcutaneous tissue disorders	Skin laxity	11	16.59 (9.05–30.41)	16.58 (153.15)	153.15	3.98 (1.97)	15.82 (8.63)
Eye disorders	Lacrimal disorder	11	7.34 (4.04–13.35)	7.34 (58.89)	58.89	2.85 (1.4)	7.2 (3.96)
Eye disorders	Amblyopia	11	7.36 (4.05–13.37)	7.36 (59.05)	59.05	2.85 (1.4)	7.21 (3.97)
Eye disorders	Blepharochalasis	11	92.53 (47.3–181.02)	92.52 (772.32)	772.32	6.17 (2.45)	71.98 (36.79)
Infections and infestations	Injection site pustule	10	10.69 (5.69–20.07)	10.69 (84.99)	84.99	3.38 (1.6)	10.38 (5.53)
Eye disorders	Extraocular muscle disorder	10	12.15 (6.46–22.85)	12.15 (98.61)	98.61	3.55 (1.68)	11.74 (6.25)
Musculoskeletal and connective tissue disorders	Head deformity	10	12.89 (6.85–24.25)	12.89 (105.41)	105.41	3.64 (1.72)	12.43 (6.6)
Nervous system disorders	Hemiplegic migraine	10	14.53 (7.71–27.38)	14.53 (120.5)	120.5	3.8 (1.79)	13.94 (7.4)
Injury, poisoning and procedural complications	VIIth nerve injury	10	63.93 (32.42–126.06)	63.93 (516.18)	516.18	5.74 (2.26)	53.44 (27.1)
Injury, poisoning and procedural complications	Transcription medication error	10	8.43 (4.5–15.8)	8.43 (63.84)	63.84	3.04 (1.43)	8.24 (4.4)
Investigations	Central nervous system function test abnormal	9	119.87 (55.72–257.88)	119.86 (771.51)	771.51	6.45 (2.14)	87.44 (40.65)
Eye disorders	Binocular eye movement disorder	9	13.51 (6.93–26.32)	13.51 (99.99)	99.99	3.7 (1.63)	13 (6.67)
Injury, poisoning and procedural complications	Product administered by wrong person	9	14.03 (7.2–27.36)	14.03 (104.35)	104.35	3.75 (1.65)	13.48 (6.92)
Blood and lymphatic system disorders	Lymphatic disorder	9	7.12 (3.68–13.79)	7.12 (46.32)	46.32	2.8 (1.2)	6.99 (3.61)
Nervous system disorders	Ophthalmic migraine	9	8.17 (4.22–15.84)	8.17 (55.24)	55.24	3 (1.31)	7.99 (4.12)
General disorders and administration site conditions	Premature aging	8	10.79 (5.33–21.83)	10.79 (68.74)	68.74	3.39 (1.37)	10.47 (5.18)
Nervous system disorders	Spasmodic dysphonia	8	10.84 (5.36–21.92)	10.83 (69.08)	69.08	3.39 (1.37)	10.51 (5.2)
General disorders and administration site conditions	Injection site alopecia	8	106.55 (47.87–237.17)	106.54 (627.32)	627.32	6.32 (1.94)	80.16 (36.01)
Injury, poisoning and procedural complications	Product administered from unauthorized provider	8	106.55 (47.87–237.17)	106.54 (627.32)	627.32	6.32 (1.94)	80.16 (36.01)
Skin and subcutaneous tissue disorders	Facial wasting	8	12.85 (6.34–26.05)	12.85 (84.04)	84.04	3.63 (1.47)	12.39 (6.11)
Musculoskeletal and connective tissue disorders	Neuropathic muscular atrophy	8	20.3 (9.93–41.47)	20.29 (137.99)	137.99	4.26 (1.67)	19.14 (9.37)
Nervous system disorders	Autonomic dysreflexia	8	21.86 (10.68–44.74)	21.86 (149.02)	149.02	4.36 (1.7)	20.52 (10.03)
Investigations	Neutralizing antibodies	8	29.06 (14.09–59.93)	29.06 (198.67)	198.67	4.74 (1.78)	26.72 (12.96)
Respiratory, thoracic and mediastinal disorders	Intranasal hypoaesthesia	8	32.37 (15.64–66.98)	32.37 (220.83)	220.83	4.88 (1.81)	29.48 (14.25)
Gastrointestinal disorders	Salivary gland disorder	8	6.38 (3.17–12.84)	6.38 (35.56)	35.56	2.65 (1.01)	6.27 (3.11)
Respiratory, thoracic and mediastinal disorders	Oropharyngeal spasm	8	7.31 (3.63–14.73)	7.31 (42.57)	42.57	2.84 (1.11)	7.16 (3.55)
Injury, poisoning and procedural complications	Eyelid contusion	8	71.03 (33.02–152.82)	71.03 (451.92)	451.92	5.87 (1.93)	58.3 (27.1)
General disorders and administration site conditions	Application site acne	8	8.09 (4.01–16.32)	8.09 (48.5)	48.5	2.98 (1.19)	7.92 (3.92)
Nervous system disorders	Vocal cord paresis	7	11.97 (5.63–25.45)	11.96 (67.8)	67.8	3.53 (1.27)	11.57 (5.44)
General disorders and administration site conditions	Injection site muscle weakness	7	117.76 (49.5–280.14)	117.76 (592.19)	592.19	6.43 (1.72)	86.32 (36.29)
Nervous system disorders	Trigeminal nerve disorder	7	7.64 (3.61–16.16)	7.64 (39.43)	39.43	2.9 (1.01)	7.48 (3.54)
Nervous system disorders	Bulbar palsy	6	10.31 (4.57–23.25)	10.31 (48.87)	48.87	3.32 (1.02)	10.02 (4.44)
Injury, poisoning and procedural complications	Oral contusion	6	11.35 (5.03–25.62)	11.35 (54.67)	54.67	3.46 (1.06)	10.99 (4.87)
Nervous system disorders	Neuralgic amyotrophy	6	11.35 (5.03–25.62)	11.35 (54.67)	54.67	3.46 (1.06)	10.99 (4.87)
General disorders and administration site conditions	Injection site muscle atrophy	6	112.82 (44.48–286.14)	112.81 (491.46)	491.46	6.39 (1.46)	83.64 (32.98)
Product issues	Product quality control issue	6	12.7 (5.62–28.72)	12.7 (62.2)	62.2	3.62 (1.11)	12.25 (5.42)
Reproductive system and breast disorders	Pelvic floor muscle weakness	6	14.75 (6.51–33.44)	14.75 (73.53)	73.53	3.82 (1.18)	14.15 (6.24)
General disorders and administration site conditions	Injection site movement impairment	6	19.57 (8.58–44.63)	19.57 (99.62)	99.62	4.21 (1.27)	18.5 (8.11)
Nervous system disorders	Radiculitis brachial	6	20.4 (8.94–46.57)	20.4 (104.06)	104.06	4.27 (1.28)	19.24 (8.43)
General disorders and administration site conditions	Injection site deformation	6	30.93 (13.38–71.51)	30.93 (158.45)	158.45	4.82 (1.38)	28.29 (12.24)
Psychiatric disorders	Compulsive lip biting	5	19.73 (8–48.69)	19.73 (83.74)	83.74	4.22 (1.02)	18.64 (7.55)
Nervous system disorders	Cervicogenic headache	5	19.98 (8.09–49.31)	19.98 (84.83)	84.83	4.24 (1.02)	18.86 (7.64)
Eye disorders	Swollen tear duct	5	20.49 (8.3–50.61)	20.49 (87.11)	87.11	4.27 (1.03)	19.32 (7.82)
Eye disorders	Lid lag	5	38.05 (15.05–96.18)	38.05 (161.2)	161.2	5.09 (1.13)	34.11 (13.5)

*Note:* An overview of adverse events (AEs) demonstrating strong disproportionality signals across 14 SOCs and their corresponding 156 Preferred Terms (PTs), as identified through established signal detection criteria. 1. Drug ineffectiveness (*n* = 22 262) and off‐label use (*n* = 11 441) were the most commonly reported PTs with the frequency of reports. 2. In the reporting odds ratio (ROR) analysis, eyebrow ptosis demonstrated the highest association (ROR = 10 060.91), trailed by facial paresis (ROR = 3303.51) and botulism (ROR = 1215.27). Additionally, neuromuscular toxicity (ROR = 533.20) and reuse of single‐use products (ROR = 258.89) showed notably elevated ROR values. 3. The *χ* ^2^ analysis revealed that blepharoptosis had the strongest statistical relationship (*χ* ^2^ = 391 328.68), followed by product preparation errors (*χ* ^2^ = 231 995.87) and reuse of single‐use products (*χ* ^2^ = 204 739.75) as the next most prominent associations. 4. Bayesian analyses provided corroborative evidence for the ROR findings, demonstrating that eyebrow ptosis (EBGM = 310.73) and botulism (EBGM = 253.76) maintained consistently elevated values across both statistical approaches.

In ROR analysis, eyebrow ptosis (SOC: skin and subcutaneous tissue disorders; *n* = 502; ROR = 10 060.91) exhibited the strongest association, followed by facial paresis (SOC: injury, poisoning, and procedural complications; *n* = 31; ROR = 3303.51) and botulism (SOC: infections and infestations; *n* = 296; ROR = 1215.27). In addition, neuromuscular toxicity (SOC: nervous system disorders; *n* = 140; ROR = 533.20) and reuse of single‐use product (SOC: injury, poisoning, and procedural complications; *n* = 1450; ROR = 258.89) produced high ROR values.

According to *χ* ^2^ values, blepharoptosis (*n* = 3140; *χ* ^2^ = 391 328.68) had the strongest statistical association, followed by product preparation error (*n* = 3174; *χ* ^2^ = 231 995.87) and reuse of single‐use product (*n* = 1450; *χ* ^2^ = 204 739.75). Bayesian approaches corroborated the findings of the ROR analysis: eyebrow ptosis (IC = 8.28; EBGM = 310.73) and botulism (IC = 7.99; EBGM = 253.76) exhibited consistently high values across both metrics.

## 4 Discussion

The analysis of 155 449 pharmacovigilance reports from the FAERS shows that BoNT‐A‐related ADRs were reported across 27 SOCs. The highest reporting frequencies were observed in general disorders and administration‐site conditions; injury, poisoning, and procedural complications; and nervous system disorders. Reports were also frequent under eye disorders and musculoskeletal and connective tissue disorders, indicating that BoNT‐A effects extend beyond the local injection site.

According to the recent ISAPS Global Survey from 2010 to 2023, BoNT‐A injection remains one of the most frequently performed nonsurgical cosmetic procedures [17]. Among the 60 622 unique case reports analyzed, 87.8% of events were reported in females—mirroring the predominance of women among recipients of BoNT‐A‐based aesthetic treatments. Among males, the incidence of BoNT‐A AEs accounted for 12.2% of the total population, which may be attributed to the smaller number of male patients receiving BoNT‐A therapy and variations in the application scope and dosage of BoNT‐A.

Studies indicate that the proportion of males undergoing cosmetic procedures has remained around 14% in recent years [17]. Male aesthetic preferences undoubtedly represent an increasingly prominent trend; however, achieving comparable rates and numbers to those observed in females will be a key direction for future development. Male and female individuals exhibit distinct physiological differences, such as greater skeletal muscle mass, higher facial vascular density, and more pronounced facial wrinkles. These anatomical variations directly influence the required dosage and efficacy of BoNT‐A injections. Literature reports that males require higher doses of BoNT‐A for cosmetic applications compared to females, and even with increased doses, their response rates remain lower than those of females [18]. Besides, studies have demonstrated that the effective dosage and duration of BoNT‐A treatment for adductor laryngeal dystonia remain consistent regardless of age or gender [19]. This further highlights the expanding applications of BoNT‐A beyond medical aesthetics into therapeutic areas, including the treatment of blepharospasm, hemifacial spasm, cervical dystonia, hyperhidrosis, and facial wrinkles [2, 3]—a potential explanation for the observed gender‐specific variations in AEs. The peak age distribution of ADRs (18–64.9 years; 37.9%) is consistent with the typical demographic seeking aesthetic BoNT‐A treatment [20]. Together, these demographic findings reinforce the need for careful safety monitoring among aesthetic BoNT‐A recipients.

The upward trend in annual ADR reports from 2004 to 2025 parallels the documented increase in global BoNT‐A use [21]. Although 45.8% of these reports originated from healthcare professionals, consumer‐initiated reports accounted for a substantial proportion (32.5%). Although most events were classified as nonserious (86.4%) and nonfatal (98.0%), the occurrence of serious outcomes such as hospitalization (4.0%) and death (2.0%) underscores the importance of prompt recognition and management of BoNT‐A‐related complications by all practitioners.

Our analysis revealed a biphasic temporal pattern of ADR onset: an initial peak within the first 30 post‐injection days and a smaller second peak after the 360‐day mark. The early peak is consistent with the established pharmacodynamics of BoNT‐A—clinical effects typically emerge within 24 h to 2 weeks of injection and persist for 3–6 months [21, 22]. The early‐onset pattern is further corroborated by preclinical studies, which indicate that most BoNT‐A formulations retain ≥ 90% potency for at least 12 weeks following injection. BoNT‐A undergoes rapid initial distribution (half‐life measured in minutes) followed by slower elimination (half‐life measured in hours), both of which are dose‐independent [23]. These kinetic phases likely contribute to the prolonged neuromuscular effects observed clinically and may help further explain the early onset of ADRs.

On the other hand, the delayed peak might stem from the cumulative neurophysiological alterations caused by repeated injections, complications that arise later due to the spread of the toxin, or the development of neutralizing antibodies leading to secondary treatment failure (described as the medication being ineffective). The extended duration of certain PTs—most notably dysphagia (7702 days), facial paralysis (6360 days), and neck pain (5928 days)—may be consistent with the mechanism of action of BoNT‐A, which involves sustained inhibition of acetylcholine release at presynaptic terminals, leading to prolonged neuromuscular blockade [2, 24]. Clinicians should therefore monitor patients for both early‐ and late‐onset ADRs following BoNT‐A administration.

Using four pharmacovigilance algorithms (PRR, EBGM, BCPNN, and ROR), we identified several strong safety signals, including eyebrow ptosis, botulism, blepharoptosis, facial paresis, and reuse of single‐use product. Eyebrow ptosis and blepharoptosis are known complications arising from unintended chemodenervation of the levator palpebrae superioris or brow elevator muscles following local toxin diffusion [25]. Anatomically, eyebrow ptosis arises from an imbalance between the frontalis (brow elevator) and the depressors of the glabellar complex (procerus, corrugator supercilii, and orbicularis oculi). In patients treated for horizontal forehead rhytides, concurrent treatment of the glabella is essential; otherwise, unopposed depressor activity may precipitate brow descent. Maintaining injection points at least 2–3 cm above the supraorbital rim or 1.5–2 cm above the brow, along with precise dose and dilution control, can reduce the risk of eyebrow ptosis. Blepharoptosis typically occurs when toxin injected near the midpupillary line at the superior orbital rim migrates through the orbital septum and weakens the levator palpebrae superioris [26, 27]. Dermatochalasis in older individuals also contributes to this complication, as these patients unconsciously recruit the frontalis to elevate the brow and eyelids; BoNT‐A injection may weaken this compensatory mechanism, thus unmasking or exacerbating ptosis. Although these effects may persist for weeks, they are generally self‐limited and can be partially managed with topical α‐adrenergic agonists (e.g., apraclonidine, naphazoline, and phenylephrine) until toxin‐induced paralysis resolves [22, 26].

Since 1987, BoNT‐A has been used therapeutically to improve facial symmetry in patients with facial palsy [28] and to reduce hyperkinetic facial movements [29]. However, several case reports record the occurrence of unintended muscle weakness following aesthetic BoNT‐A injection [30, 31]. Such events are generally attributed to excessive dosing or diffusion of toxin into adjacent, nontarget muscles [32]. The results of our analysis are consistent with these findings: facial paresis emerged as a strong safety signal. At present, a clearing threshold separating therapeutic effect from local toxicity is lacking; risk mitigation therefore depends on injector experience and precise technique.

Other safety signals—reuse of single‐use product and product preparation error—are directly associated with injector practice and product handling, underscoring the importance of procedural accuracy in BoNT‐A treatments. Improving injection proficiency, adhering to aseptic nontouch technique, and selecting high‐quality products are key to reducing procedural risk.

Otherwises, experimental evidence indicates that BoNT‐A undergoes retrograde axonal transport to brainstem nuclei in murine models, where it retains catalytic activity, is released within the facial nucleus, and preferentially targets central cholinergic synapses [33]. This trans‐synaptic action may modulate spinal and cortical circuitry [34]. Although the clinical significance of central transport in humans remains debated, central nervous system symptoms—including headache, dizziness, cognitive changes, and, rarely, encephalopathy or coma (typically in the context of systemic toxicity)—have been reported following BoNT‐A exposure. Botulism, the most severe manifestation of systemic toxicity, often presents initially with intense headache and fatigue, progressing to descending flaccid paralysis [35].

Beyond central effects, peripheral neuromuscular manifestations—including muscle weakness and sensory disturbances—have been frequently reported following BoNT‐A exposure. BoNT‐A inhibits acetylcholine release at the presynaptic membrane, resulting in dose‐dependent muscle denervation and weakness. While effects are most pronounced near the injection site, hematogenous dissemination may affect noninjected muscles, leading to generalized weakness, dyspnea, and even coma [35]. Our findings of prolonged dysphagia, facial paralysis, and neck pain are consistent with these reported effects.

The widespread distribution of cholinergic neuromuscular junctions renders multiple organ systems, including ocular tissues, susceptible to BoNT‐A effects. The effects on ocular tissues have been attributed to the action of BoNT‐A on autonomic pathways regulating ocular function. BoNT‐A inhibits acetylcholine release from preganglionic and postganglionic parasympathetic terminals and sympathetic ganglia, and systemic intoxication can lead to fixed, mid‐dilated pupils. Mydriasis may further result from BoNT‐A uptake by parasympathetic neurons at the ciliary ganglion or the neuromuscular junction of the iris sphincter [36, 37]. Pupillary changes may therefore serve as an early clinical sign of systemic toxicity. Notably, ocular ADRs were among the most frequently reported SOCs in our analysis. One of the main contributors to these ocular complications may be injection technique: high‐volume injections near the lateral canthus or tarsal plate can impair lid closure and exacerbate dry eye by reducing tear production and increasing evaporation. Conversely, BoNT‐A injection into the medial eyelid can alleviate dry eye by impairing lacrimal drainage. While injections lateral to the brow, placed 1 cm above the orbital rim, have been shown to prevent unwanted orbicularis oculi paralysis, a total dosage exceeding 50 units per session may elevate complication risk [38, 39]. Periorbital injections have also been associated with acute angle‐closure glaucoma, retinal detachment, blurred vision, and corneal exposure [37].

At low doses, BoNT‐A effects remain largely confined to the target muscle; however, higher doses may increase the likelihood of diffusion to adjacent muscles, including across fascial planes [35]. Clinical reports have described unintended effects following injection at various sites, including laryngeal adverse effects after periocular injection for treatment of blepharospasm [40]. In the lower face and cervical region, BoNT‐A spread has been associated with functional impairments such as difficulty opening the mouth, dysphonia, dysphagia, or worsening dystonia [41, 42, 43] In addition, diffusion of BoNT‐A to the bladder neck can reduce smooth muscle contractility, potentially causing retrograde ejaculation and decreased ejaculate volume in male patients [44]. When injections are administered near vascular structures, systemic anticholinergic effects—culminating in iatrogenic botulism—may ensue.

Iatrogenic botulism is a rare but severe condition characterized by descending flaccid paralysis with potential progression to respiratory failure, bulbar palsy, autonomic dysfunction, and dysphagia [44, 45]. Botulism is among the most severe adverse effects of BoNT‐A injection, with patients often requiring hospitalization [46]. Management of this condition includes aggressive supportive care, early intubation and mechanical ventilation, and administration of equine‐derived botulinum antitoxin [47].

### 4.1 Limitations

This retrospective, database‐derived study has several limitations. First, the FAERS database is susceptible to reporting biases, including under‐reporting, stimulated reporting, and incomplete clinical information (e.g., missing data on injection dose, dilution, and technique). Second, disproportionality analysis identifies statistical associations, not causal relationships. Third, the generalizability of these findings may be constrained by the predominance of reports originating from the United States of America.

Standardized data collection protocols would help address these limitations in future prospective studies. Linkage of pharmacovigilance data with electronic health records could provide additional clinical context. Finally, multinational collaborative efforts would be valuable in validating these findings across diverse populations and healthcare settings.

## 5 Conclusion

This pharmacovigilance study, drawing on FAERS data from 2004 to 2025, delineates a comprehensive ADR profile for BoNT‐A. ADR onset followed a biphasic temporal pattern, initially peaking within 30 days after the injection and again beyond the 360‐day mark. Strong safety signals were detected for eyebrow ptosis, botulism, blepharoptosis, and facial paresis. Reuse of single‐use products was also identified as a safety signal. These findings suggest that factors such as injection technique, dose control, and knowledge of functional anatomy may influence the occurrence of ADRs. Accordingly, careful monitoring of patients during both early and late post‐treatment phases may be warranted to ensure timely identification and mitigation of ADRs. Continued postmarketing surveillance and targeted educational initiatives are needed for improving the safety profile of BoNT‐A formulations in both aesthetic and therapeutic applications.

## Author Contributions

Jiaxu Gu wrote the first draft of the manuscript and prepared tables together with Yue Sun. Kexin Chen, Jieyi Wang, Bingcheng Lu, and Hongqiang Xie collected and reviewed published articles. Xiaoming Liu, Cong Huang, Xingling Jian, and Bo Yu supervised the study and provided funding support. All authors reviewed and approved the final manuscript. Jiaxu Gu and Yue Sun contributted equally to this manuscript.

## Funding

This study was supported by Guangdong Basic and Applied Basic Research Foundation (2025A1515010947, 2024A1515220018), Shenzhen Sanming Project (No. SZSM202311029), Shenzhen Key Medical Discipline Construction Fund (SZXK040), Shenzhen High‐level Hospital Construction Fund, Peking University Shenzhen Hospital Scientific Research Fund (KYQD2024378, KYQD2021052), and National Natural Science Foundation of China (81803138).

## Ethics Statement

Ethics approval was waived for this study because no patients' data were reported.

## Consent

The authors have nothing to report.

## Conflicts of Interest

The authors declare no conflicts of interest.

## Data Availability

The data that support the findings of this study are available from the corresponding author upon reasonable request.

## References

[1] R. Kumar and B. R. Singh , “Botulinum Toxin: A Comprehensive Review of Its Molecular Architecture and Mechanistic Action,” International Journal of Molecular Sciences 26, no. 2 (2025): 777. 39859491 10.3390/ijms26020777 PMC11766063

[2] S. Choudhury , M. R. Baker , S. Chatterjee , and H. Kumar , “Botulinum Toxin: An Update on Pharmacology and Newer Products in Development,” Toxins 13, no. 1 (2021): 58. 33466571 10.3390/toxins13010058 PMC7828686

[3] L. A. Dessy , N. Fallico , M. Mazzocchi , and N. Scuderi , “Botulinum Toxin for Glabellar Lines: A Review of the Efficacy and Safety of Currently Available Products,” American Journal of Clinical Dermatology 12, no. 6 (2011): 377–388. 21877763 10.2165/11592100-000000000-00000

[4] E. Yiannakopoulou , “Serious and Long‐Term Adverse Events Associated With the Therapeutic and Cosmetic Use of Botulinum Toxin,” Pharmacology 95, no. 1–2 (2015): 65–69. 25613637 10.1159/000370245

[5] I. R. Edwards and J. K. Aronson , “Adverse Drug Reactions: Definitions, Diagnosis, and Management,” Lancet 356, no. 9237 (2000): 1255–1259. 11072960 10.1016/S0140-6736(00)02799-9

[6] D. Zargaran , F. Zoller , A. Zargaran , et al., “Complications of Cosmetic Botulinum Toxin a Injections to the Upper Face: A Systematic Review and Meta‐Analysis,” Aesthetic Surgery Journal 42, no. 5 (2022): NP327–NP336. 35178552 10.1093/asj/sjac036 PMC9005453

[7] É. P. Di Santis , S. H. Hirata , G. M. Di Santis , and S. Yarak , “Adverse Effects of the Aesthetic Use of Botulinum Toxin and Dermal Fillers on the Face: A Narrative Review,” Anais Brasileiros de Dermatologia 100, no. 1 (2025): 87–103. 39616095 10.1016/j.abd.2024.04.007 PMC11745296

[8] R. J. Yu , M. S. Krantz , E. J. Phillips , and C. A. Stone , “Emerging Causes of Drug‐Induced Anaphylaxis: A Review of Anaphylaxis‐Associated Reports in the FDA Adverse Event Reporting System (FAERS),” Journal of Allergy and Clinical Immunology: In Practice 9, no. 2 (2021): 819–829.e2. 32992044 10.1016/j.jaip.2020.09.021 PMC7870524

[9] Y. Wang , B. Zhao , H. Yang , and Z. Wan , “A Real‐World Pharmacovigilance Study of FDA Adverse Event Reporting System Events for Sildenafil,” Andrology 12, no. 4 (2024): 785–792. 37724699 10.1111/andr.13533

[10] X. Zhang and Y. Wang , “The Pharmacovigilance Analysis of Adverse Events of Erlotinib Based on the Open Access FAERS Database,” MedBA Medicine 1, no. 1 (2026): 9–18.

[11] Y. Xu , B. Zhao , and L. He , “Adverse Drug Reaction Signals Mining Comparison of Amiodarone and Dronedarone: A Pharmacovigilance Study Based on FAERS,” Frontiers in Pharmacology 15 (2024): 1438292. 39502527 10.3389/fphar.2024.1438292 PMC11534617

[12] C. Zhong , Q. Zheng , B. Zhao , and T. Ren , “A Real‐World Pharmacovigilance Study Using Disproportionality Analysis of United States Food and Drug Administration Adverse Event Reporting System Events for Vinca Alkaloids: Comparing Vinorelbine and Vincristine,” Expert Opinion on Drug Safety 23, no. 11 (2024): 1427–1437. 39340205 10.1080/14740338.2024.2410436

[13] S. Zhao , Y. Wang , X. Deng , X. Chen , and Z. Lu , “Analysis of ADR Reports of Cetuximab Based on the FDA Adverse Event Reporting System Database,” Scientific Reports 15, no. 1 (2025): 4104. 39901061 10.1038/s41598-025-88838-z PMC11790939

[14] B. Zhao , Y. Fu , S. Cui , X. Chen , S. Liu , and L. Luo , “A Real‐World Disproportionality Analysis of Everolimus: Data Mining of the Public Version of FDA Adverse Event Reporting System,” Frontiers in Pharmacology 15 (2024): 1333662. 38533254 10.3389/fphar.2024.1333662 PMC10964017

[15] H. Yang , Z. Wan , M. Chen , X. Zhang , W. Cui , and B. Zhao , “A Real‐World Data Analysis of Topotecan in the FDA Adverse Event Reporting System (FAERS) Database,” Expert Opinion on Drug Metabolism & Toxicology 19, no. 4 (2023): 217–223. 37243615 10.1080/17425255.2023.2219390

[16] W. He , Y. Wang , and K. Chen , “A Real‐World Pharmacovigilance Study of FDA Adverse Event Reporting System Events for Diazepam,” Frontiers in Pharmacology 15 (2024): 1278442. 38327980 10.3389/fphar.2024.1278442 PMC10847318

[17] L. Triana , R. M. Palacios Huatuco , G. Campilgio , and E. Liscano , “Trends in Surgical and Nonsurgical Aesthetic Procedures: A 14‐Year Analysis of the International Society of Aesthetic Plastic Surgery‐ISAPS,” Aesthetic Plastic Surgery 48, no. 20 (2024): 4217–4227. 39103642 10.1007/s00266-024-04260-2

[18] T. C. Keaney and T. S. Alster , “Botulinum Toxin in Men: Review of Relevant Anatomy and Clinical Trial Data,” Dermatologic Surgery: Official Publication for American Society for Dermatologic Surgery 39, no. 10 (2013): 1434–1443. 10.1111/dsu.12302 24090254

[19] S. Vasconcelos , H. Birkent , M. G. Sardesai , A. L. Merati , and A. D. Hillel , “Influence of Age and Gender on Dose and Effectiveness of Botulinum Toxin for Laryngeal Dystonia,” Laryngoscope 119, no. 10 (2009): 2004–2007. 19572275 10.1002/lary.20564

[20] F. Farashi , M. Ghoncheh , M. R. Ghasemian Moghaddam , and Z. Soroosh , “Evaluation of People's Satisfaction With Botulinum Toxin Injection for Facial Rejuvenation Based on Age,” Journal of Cosmetic Dermatology 23, no. 7 (2024): 2380–2385. 38500014 10.1111/jocd.16289

[21] G. J. Jeong , J. H. Kim , H. J. Oh , K. Y. Park , and S. J. Seo , “Potency and Persistence of Reconstituted Botulinum Neurotoxin Type a: Mouse IP LD50 Assay,” Dermatologic Surgery 46, no. 10 (2020): e78–e81. 31356438 10.1097/DSS.0000000000002070

[22] B. K. Satriyasa , “Botulinum Toxin (Botox) a for Reducing the Appearance of Facial Wrinkles: A Literature Review of Clinical Use and Pharmacological Aspect,” Clinical, Cosmetic and Investigational Dermatology 12 (2019): 223–228. 31114283 10.2147/CCID.S202919 PMC6489637

[23] L. Simpson , “The Life History of a Botulinum Toxin Molecule,” Toxicon: Official Journal of the International Society on Toxinology 68 (2013): 40–59. 23518040 10.1016/j.toxicon.2013.02.014

[24] I. Kao , D. B. Drachman , and D. L. Price , “Botulinum Toxin: Mechanism of Presynaptic Blockade,” Science 193, no. 4259 (1976): 1256–1258. 785600 10.1126/science.785600

[25] A. Borba , S. Matayoshi , and M. Rodrigues , “Avoiding Complications on the Upper Face Treatment With Botulinum Toxin: A Practical Guide,” Aesthetic Plastic Surgery 46, no. 1 (2022): 385–394. 34341857 10.1007/s00266-021-02483-1 PMC8328485

[26] A. Redaelli and R. Forte , “How to Avoid Brow Ptosis After Forehead Treatment With Botulinum Toxin,” Journal of Cosmetic and Laser Therapy 5, no. 3–4 (2003): 220–222. 14741838 10.1080/14764170310023254

[27] M. Kassir , M. Gupta , H. Galadari , et al., “Complications of Botulinum Toxin and Fillers: A Narrative Review,” Journal of Cosmetic Dermatology 19, no. 3 (2020): 570–573. 31889407 10.1111/jocd.13266

[28] C. de Sanctis Pecora and D. Shitara , “Botulinum Toxin Type a to Improve Facial Symmetry in Facial Palsy: A Practical Guideline and Clinical Experience,” Toxins 13, no. 2 (2021): 159. 33670477 10.3390/toxins13020159 PMC7923088

[29] G. Goodman , “Botulinum Toxin for the Correction of Hyperkinetic Facial Lines,” Australasian Journal of Dermatology 39, no. 3 (1998): 158–163. 9737041 10.1111/j.1440-0960.1998.tb01272.x

[30] M. P. Auada Souto and L. R. M. Souto , “An Unusual Adverse Event of Botulinum Toxin Injection in the Lower Face,” Journal of Cosmetic Dermatology 20, no. 5 (2021): 1381–1384. 33249739 10.1111/jocd.13869

[31] X. Zuo and D. Yang , “Acquired Bilateral Inferior Oblique Muscle Paralysis Following Facial Injections of Botulinum Toxin,” European Journal of Ophthalmology 35, no. 6 (2025): NP42–NP45. 10.1177/11206721251367813 40802057

[32] H. Sundaram , M. Signorini , S. Liew , et al., “Global Aesthetics Consensus: Botulinum Toxin Type A–Evidence‐Based Review, Emerging Concepts, and Consensus Recommendations for Aesthetic Use, Including Updates on Complications,” Plastic and Reconstructive Surgery 137, no. 3 (2016): 518e–529e. 10.1097/01.prs.0000475758.63709.23 PMC5242214 26910696

[33] M. Caleo , M. Spinelli , F. Colosimo , et al., “Transynaptic Action of Botulinum Neurotoxin Type a at Central Cholinergic Boutons,” Journal of Neuroscience 38, no. 48 (2018): 10329–10337. 30315128 10.1523/JNEUROSCI.0294-18.2018 PMC6596210

[34] G. Abbruzzese and A. Berardelli , “Neurophysiological Effects of Botulinum Toxin Type a,” Neurotoxicity Research 9, no. 2–3 (2006): 109–114. 16785106 10.1007/BF03033927

[35] J. Ramirez‐Castaneda , J. Jankovic , C. Comella , K. Dashtipour , H. H. Fernandez , and Z. Mari , “Diffusion, Spread, and Migration of Botulinum Toxin,” Movement Disorders: Official Journal of the Movement Disorder Society 28, no. 13 (2013): 1775–1783. 23868503 10.1002/mds.25582

[36] E. Hanna , L. Xing , J. H. Taylor , and V. Bertucci , “Role of Botulinum Toxin a in Improving Facial Erythema and Skin Quality,” Archives of Dermatological Research 314, no. 8 (2022): 729–738. 34519860 10.1007/s00403-021-02277-0

[37] S. Akkaya , H. K. Kökcen , and T. Atakan , “Unilateral Transient Mydriasis and Ptosis After Botulinum Toxin Injection for a Cosmetic Procedure,” Clinical Ophthalmology 9 (2015): 313–315. 25709394 10.2147/OPTH.S76054 PMC4334348

[38] J. C. Tsai , “Acute Angle Closure Following Periorbital Botulinum Toxin Injection in a Patient With Retinitis Pigmentosa,” Taiwan Journal of Ophthalmology 7, no. 2 (2017): 104–107. 29018766 10.4103/tjo.tjo_41_17 PMC5602147

[39] R. W. Ho , P. C. Fang , C. H. Chang , Y. P. Liu , and M. T. Kuo , “A Review of Periocular Botulinum Neurotoxin on the Tear Film Homeostasis and the Ocular Surface Change,” Toxins 11, no. 2 (2019): 66. 30678375 10.3390/toxins11020066 PMC6409927

[40] M. E. Northington and C. C. Huang , “Dry Eyes and Superficial Punctate Keratitis: A Complication of Treatment of Glabelar Dynamic Rhytides With Botulinum Exotoxin a,” Dermatologic Surgery 30, no. 12 Pt 2 (2004): 1515–1517. 15606828 10.1111/j.1524-4725.2004.30556.x

[41] H. Witmanowski and K. Błochowiak , “The Whole Truth About Botulinum Toxin – A Review,” Postepy Dermatologii I Alergologii 37, no. 6 (2020): 853–861. 33603602 10.5114/ada.2019.82795 PMC7874868

[42] H. L. P. Peng and J. H. Peng , “Complications of Botulinum Toxin Injection for Masseter Hypertrophy: Incidence Rate From 2036 Treatments and Summary of Causes and Preventions,” Journal of Cosmetic Dermatology 17, no. 1 (2018): 33–38. 29250900 10.1111/jocd.12473

[43] A. Camões‐Barbosa , I. M. Ribeiro , and L. Medeiros , “Contralateral Upper Limb Weakness Following Botulinum Toxin a Injection for Poststroke Spasticity,” Acta Médica Portuguesa 33, no. 11 (2020): 761–764. 31759399 10.20344/amp.11503

[44] S. M. Comtesse , F. Tavassoli , and B. Jabbari , “Botulinum Toxin Treatment of Lingual Dystonia, Oromandibular Dystonia and Bruxism‐a Review and Update,” Toxicon: Official Journal of the International Society on Toxinology 266 (2025): 108503. 40714113 10.1016/j.toxicon.2025.108503

[45] R. Caremel , F. Courtois , K. Charvier , A. Ruffion , and N. M. Journel , “Side Effects of Intradetrusor Botulinum Toxin Injections on Ejaculation and Fertility in Men With Spinal Cord Injury: Preliminary Findings,” BJU International 109, no. 11 (2012): 1698–1702. 21981647 10.1111/j.1464-410X.2011.10639.x

[46] A. K. Rao , J. Sobel , K. Chatham‐Stephens , and C. Luquez , “Clinical Guidelines for Diagnosis and Treatment of Botulism, 2021,” MMWR – Recommendations and Reports 70, no. 2 (2021): 1–30. 10.15585/mmwr.rr7002a1 PMC8112830 33956777

[47] M. Pohanka , “Botulinum Toxin as a Biological Warfare Agent: Poisoning, Diagnosis and Countermeasures,” Mini‐Reviews in Medicinal Chemistry 20, no. 10 (2020): 865–874. 32108007 10.2174/1389557520666200228105312

